# Knowledge of Human Cytomegalovirus Infection and Prevention in Pregnant Women: A Baseline, Operational Survey

**DOI:** 10.1155/2017/5495927

**Published:** 2017-07-31

**Authors:** Maria Mazzitelli, Mariella Micieli, Carmela Votino, Federica Visconti, Paola Quaresima, Alessio Strazzulla, Carlo Torti, Fulvio Zullo

**Affiliations:** ^1^Unit of Infectious and Tropical Diseases, Department of Medical and Surgical Sciences, “Magna Graecia” University of Catanzaro, Viale Europa, 88100 Catanzaro, Italy; ^2^Unit of Obstetrics and Gynecology, Department of Medical and Surgical Sciences, “Magna Graecia” University of Catanzaro, Viale Europa, 88100 Catanzaro, Italy

## Abstract

Currently, the only efficient way to prevent human Cytomegalovirus (HCMV) infection in pregnancy is primary prophylaxis through hygienic measures. So, we evaluated knowledge of HCMV and its prevention in a group of pregnant women. An anonymous questionnaire with multiple-choice answers was administered to all pregnant women who were followed up at the Obstetrics and Gynecology Unit of “Pugliese-Ciaccio Hospital,” a third-level hospital in Catanzaro (Southern Italy), from November 2015 to March 2016. Previously prescribed serology results for HCMV were also evaluated. Three hundred and fifty women participated in the study and the results clearly demonstrated that knowledge of pregnant women about HCMV is poor. Moreover, prescribed screening procedures need to be optimized, since one out of three pregnant women has not been tested for HCMV or the screening was not performed adequately. For this reason, it is important to implement informative campaign in both pregnant women and providing physicians.

## 1. Background

Human Cytomegalovirus (HCMV) is a major cause of intrauterine and congenital infection [1]. As a consequence of a primary infection during pregnancy, newborns may be affected by several conditions, such as hearing loss, mental retardation, and motor and cognitive impairment [2, 3]. The most dangerous consequences for the newborns occur when the mother is primarily infected in the earlier period of pregnancy. Moreover, the majority of infections occur in the first trimester. Available data show that main ways of transmission are contact with children or sexual activity [4]. Since a vaccine is not actually available to avoid primary infection, prevention through hygienic measures is the only way to prevent infections. These include mainly avoiding strict contacts with children (in particular their saliva or urine) and washing hands accurately after this kind of contacts [5].

With this objective in mind, screening to identify seronegative women at risk and counselling of these women are fundamental. Indeed, a recent randomized study demonstrated that the aforementioned measures significantly prevented maternal infections and negative consequences for the newborns [6].

Through a questionnaire-based survey, we aimed at assessing level of knowledge of HCMV infection and its consequences and prevention in pregnant women in order to estimate the need of information campaigns, with the final aim of reducing primary infections in pregnancy.

## 2. Population and Methods

This study was conducted through a questionnaire administrated to all pregnant women who were followed up at the Obstetrics and Gynecology Unit of “Pugliese-Ciaccio Hospital,” a third-level public hospital in Catanzaro (Southern Italy), from November 2015 to March 2016.

Patients were asked, through expression of verbal consent, to participate in the study at their first consultation in our outpatient clinic. After medical consult, they could refuse to participate in the survey, signing a form to opt out. This form also contained information about HCMV.

An anonymous questionnaire was composed of multiple-choice answers as illustrated in Table 1. Anonymity was guaranteed to provide more reliable answers. Predefined multiple-choice answers were used to get more objective measures of patient knowledge and beliefs on this subject.

Questions #1 and #3 aimed at verifying whether women knew HCMV and whether this could have been dangerous if contracted into pregnancy or not. Question #2 wanted to explore whether pregnant women had been screened for HCMV infection or not. Lastly, question #4 was about behaviours that pregnant women thought should have been practiced to avoid HCMV infection.

The questionnaire was administered in a separated setting, from another person who did not know patient identity, independently from consultant physicians.

To provide indirect evidence on the level of knowledge among physicians, we assessed in the study population whether serological test for HCMV was already performed. In line with this purpose, we analysed all the documentation that patients brought to the consult, recording also the number of subjects who did not exhibit any documentation.

We also recorded age, nationality (Italian, European, and extra-European) and town of origin (Catanzaro, Cosenza, Crotone, Reggio Calabria, and Vibo Valentia), parity, gestational age, level of education (illiterate, primary or secondary school, and university degree), and profession (housewife, employed, and freelancer).

Pregnant women were stratified according to their level of education in two groups: high level of education (women who attended at least secondary school or were graduated) and low level of education (women who attended primary school or did not attend any school).

After completing the questionnaire, pregnant women received appropriate counselling on vertically transmitted infections (including HCMV) by the providing physicians. A leaflet with HCMV information was also distributed to the patients.

All the analyses were performed with SPSS statistical software.

## 3. Results

### 3.1. Characteristics of the Study Population

Three hundred and fifty pregnant women were proposed to fill in the questionnaire and all of them accepted. Patient characteristics are illustrated in Table 2. Median age was 31 years (standard deviation (SD): 4.24). The majority of women came from Italy (336/350, 96%), mainly from Catanzaro province. 65% (229/350) were nulliparous. Mean gestational age was 31 weeks (SD: 6.36).

Regarding level of education, 199/350 (56.8%) women completed a secondary school, 100/350 (28.6%) women were graduated, and 49/350 (14%) women attended only primary school or did not attend any schools (2/350, 0.6%). So, according to the stratification by level of education reported in Population and Methods, 299/350 (85.4%) women had a high level of education and 51/350 (14.6%) women had a low level of education.

As for the profession, most women were housewives (183/350, 52.3%) or employed (108/350, 30.9%). Among women who were housewives, 143/183 (78.1%) presented a high level of education, while among women who were employed or freelancers, 156/167 (93.4%) had a high level of education; difference between the two groups was statistically significant (*p* < 0.001).

### 3.2. Answers to the Questionnaire

Regarding question #1, 195/350 (55.7%) women answered to have knowledge of HCMV infection, 152/350 (43.4%) women did not ever hear about HCMV, and 3/350 (0.9%) women did not provide any answers.

Regarding question #2, 251/350 (71.7%) women reported to have already been screened for HCMV, 49/350 (14%) women reported that they have not been screened, and 50/350 (14.3%) women did not remember whether tests for HCMV infection had been performed or not.

For question #3, 271/350 (77.4%) women correctly thought that HCMV infection is dangerous if acquired during pregnancy. By contrast, 31/350 (8.8%) women thought that HCMV infection is not a problem if acquired during pregnancy and 48/350 (13.8%) did not know whether HCMV infection was dangerous if acquired during pregnancy.

**Figure 1:**
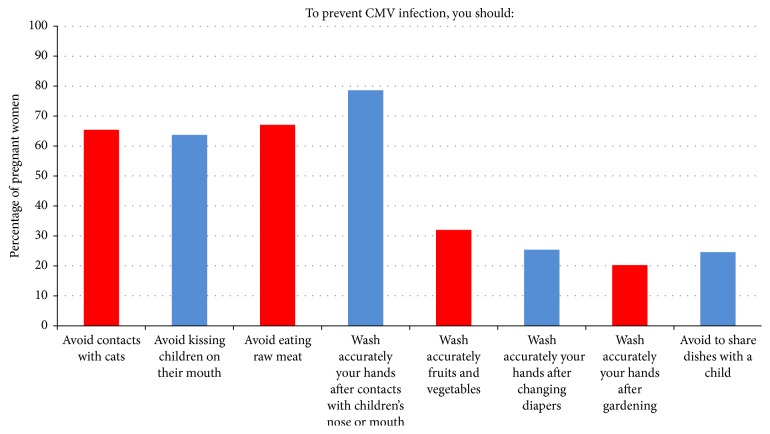


Answers to question #4 are depicted in Figure 1. Among 350 women, 326 (93.1%) women made at least one mistake. In particular, for prevention of HCMV infection, women mistakenly thought that contact with cats should be avoided (229/350, 65.4%), eating raw meat should be avoided (223/350, 63.7%), vegetables and fruits should be accurately washed (235/350, 67.1%), and cleaning hands after gardening has to be recommended (275/350, 78.6%). By contrast, the following percentages of women correctly thought that kissing children should be avoided (112/350, 32%), washing hands after contact with children's mouth or nose (89/350, 25.4%) or after changing diapers (71/350, 20.3%) should be done, and sharing dishes with a child should be avoided (86/350, 24.6%).

### 3.3. Self-Reported Serology for HCMV and Results Actually Shown by Women at Consultation

To get further insights into the level of knowledge in terms of consciousness of the tests done, we evaluated whether women who reported to have been tested were able to demonstrate the results (tested and more informed) and those who reported that they were not been tested were able to do so (tested but less informed).

Overall, 195/350 (55.7%) women exhibited serology results for HCMV and 251/350 (71.7%) reported to have been screened for HCMV. In detail, among these 251 women who reported to have been screened, 81 (32.3%) did not exhibit any results of previous tests at clinical consultation. By contrast, 170 women (67.7%) exhibited HCMV serology tests with the following results: 23/170 (13.5%) women were screened only using IgG which were positive, 104/170 (61.2%) had a serology of previous HCMV infection (with both positive IgG and negative IgM), 40/170 (23.5%) were seronegative (with both negative IgG and negative IgM), and 3/170 (1.8%) had both IgG positive and IgM positive HCMV. For the same question, among 49 women who declared to have never been screened for HCMV, 39 (79.6%) did not exhibit any results of previous tests at clinical consultation, while 10 (20.4%) exhibited HCMV serology tests with the following results: one woman was screened only using HCMV IgG which was positive, five women presented IgG positive HCMV and IgM negative HCMV, and four were seronegative with both IgG negative and IgM negative HCMV. Lastly, among 50 women who declared at question #2 that they did not remember whether tests for HCMV infection had been performed or not, 35/50 (70%) did not exhibit any result of previous tests at clinical consultation, while 15/35 (30%) exhibited test results showing that 1/50 (2%) had IgG positive HCMV, 12/50 (20%) had IgG positive and IgM negative HCMV, and 2/50 (4%) were seronegative with both IgG negative and IgM negative HCMV.

### 3.4. Knowledge of HCMV by Level of Education and Number of Pregnancies

Knowledge of HCMV preventive measures was poor both for women with low level of education and for those with high level of education. Indeed, more than 90% of women demonstrated incomplete knowledge of HCMV infection, since they made at least one mistake in answering the questionnaire's questions [280/299 (93.6%) women with high level of education compared with 48/51 (94.1%) women with low level of education; *p* = 0.03]. Interestingly, no statistically significant differences (*p* = 0.4) were found comparing the mean values of wrong answers between women who were at their first pregnancy (mean: 4.3; SD: 2.2) and those who were already mothers (mean: 4.04; SD: 2.3). However, a statistically significant difference was found considering percentages of women who responded correctly to all answers [26/299 (8.7%) women with high level of education compared with 1/51 (1.9%) women with low level of education; *p* < 0.001].

## 4. Discussion

No treatments are available for primary HCMV infection during pregnancy. Even results of observational studies [7] and randomized controlled trial about the use of hyperimmune globulin [8] are conflicting. For these reasons, hygienic measures are the only effective means to prevent both HCMV infection during pregnancy and complications to the newborns. Indeed, a recent randomized trial demonstrated that an informative intervention in HCMV seronegative women reduced the rate of new infections and complications [5].

So, we decided to investigate level of knowledge of pregnant women in order to evaluate the need of informative campaigns. Results of our study are quite alarming because about 44% of interviewed women did not have enough knowledge of HCMV running the risk of acquiring it during pregnancy. Moreover, about 23% of the interviewed women did not know that HCMV infection might have dangerous consequences for the newborn. Misleading information was also reported, since more than 65% of women thought that some hygienic measures (such as avoiding eating raw meat and contact with cats) should be applied to prevent HCMV infection. This clearly indicates confusion between HCMV and toxoplasmosis.

It is important to note that, even considering the best-case scenario of 210/350 (60%) women tested (including both those who declared to have been tested and those who showed results of the serology test), the test was omitted in a large percentage (40%) of women until late in pregnancy (mean gestational age: 31 weeks). This evidence reflects both lack of knowledge (not only among pregnant women but also among provider physicians) and lack of reimbursement of these analyses by the Italian National Health System. The fact that women were misinformed is emphasized by the lack of concordance between self-reported testing for HCMV and availability of results at consultation. Indeed, 96/350 (27.4%) women reported to have been tested but did not show the results or* vice versa* did not know whether they have been tested but actually they were tested because they had the results. This further complicates the management of patients, leading to the risk of useless repetition of the test or omission of the test in those needing it.

Lastly, we aimed at exploring possible correlates for the lack of knowledge. When level of education was taken into account, even though HCMV knowledge was higher in better educated women, all had scarce understanding of the problem. Also, women at their first pregnancy and pluriparous women provided similar numbers of wrong answers. This finding indicates the need to implement informative campaigns in the general population of pregnant women.

This study presents several limitations. First, the number of subjects was low and limited in a short period of time. Second, given the lack of reimbursement of the test by the Italian National Health System, it is impossible to infer whether the test was not prescribed by physicians because they underestimated the problem or for administrative reasons. Anyways, given the demonstration of a significant effect of counselling and prevention measures [6], both barriers (lack of information and lack of reimbursement) should be amended. Third, we may have underestimated test prescription because women may have simply forgotten to bring the test at consultation even though they have received it. Thus, results about level of information and consciousness of the problem in the pregnant women are more significant than those on the actual prescription of the test.

## 5. Conclusions

The results of this survey indicate the need of counselling women to prevent primary infection by HCMV during pregnancy and also support recommendations set by the American College of Obstetrics and Gynecologists (ACOG) that pregnant women should be educated about HCMV prevention [9]. Informative campaigns are needed not only for women at their first pregnancy but also for those who are already mothers. We suggest that it could be useful to inform girls in advance, probably during secondary school. This kind of intervention helps to reduce primary HCMV infections and their effects during pregnancy. Last but not least, given the demonstration of a clear benefit of this intervention [6], HCMV serology test during pregnancy should be included in the list of exams reimbursed by the Italian National Health System, and it should be recommended by guidelines.

## Figures and Tables

**Table 1:** Questions and answers included in the multiple-choice questionnaire.

(1) Do you know human Cytomegalovirus (HCMV) infection?
(a) Yes
(b) No
(c) Not answer

(2) Did you undergo to the screening for this virus in your pregnancy?
(a) Yes
(b) No
(c) I do not remember

(3) Is HCMV infection dangerous if contracted during pregnancy?
(a) Yes
(b) No
(c) Not Answer

(4) To avoid HCMV infection, you should (check one or more right answers):
(a) Avoid contact with cats
(b) Avoid kissing children on their mouth
(c) Avoid eating raw meat
(d) Wash accurately your hands after contacts with children's
nose or mouth
(e) Wash accurately fruits and vegetables
(f) Wash accurately your hands after changing diapers
(g) Wash accurately your hands after gardening
(h) Avoid to share dishes with a child

Possible answers to question #4 were eight: four were right and four were wrong. The right ones were related to prevention of HCMV infection in pregnancy (avoiding contacts with children, hands cleaning after contacts with children's saliva, hands cleaning after changing child's diaper, and avoiding to share dishes with children). By contrast, the wrong ones were related to consolidated practices for toxoplasmosis prevention in pregnancy (avoiding cats, avoiding eating raw meat, washing fruits and vegetables before eating, and hands cleaning after gardening). These latter four options were inserted as confounder.

**Table 2:** Characteristics of the enrolled women (*N* = 350).

Characteristic	*N* (%)
*Nationality*	
(i) Italian	(i) 336 (96)
(ii) European	(ii) 13 (3.7)
(iii) Extra-European	(iii) 1 (0.3)

*Home Italian town*	
(i) Catanzaro	(i) 262 (74.8)
(ii) Cosenza	(ii) 16 (4.6)
(iii) Crotone	(iii) 32 (9.1)
(iv) Reggio Calabria	(iv) 9 (2.5)
(v) Vibo Valentia	(v) 18 (5)

*Number of previous pregnancies*	
(i) 0	(i) 229 (65.4)
(ii) 1	(ii) 92 (26.3)
(iii) 2	(iii) 26 (7.4)
(iv) 3	(iv) 2 (0.6)
(v) 6	(v) 1 (0.3)

*Level of education*	
(i) None	(i) 2 (0.6)
(ii) Primary school	(ii) 49 (14)
(iii) Secondary school	(iii) 199 (56.9)
(iv) Graduation	(iv) 100 (28.6)

*Occupation*	
(i) Housewife	(i) 183 (52.3)
(ii) Employed	(ii) 108 (30.9)
(iii) Freelancer	(iii) 59 (16.8)
